# Metastatic Melanoma Presenting as Intussusception in an 80-Year-Old Man: A Case Report

**DOI:** 10.1155/2013/672816

**Published:** 2013-03-04

**Authors:** Sarah Alghamdi, Yumna Omarzai

**Affiliations:** Department of Pathology, Mount Sinai Medical Center, Miami Beach, FL 33140, USA

## Abstract

Malignant melanoma of the gastrointestinal tract is an uncommon neoplasm that could be primary or metastatic. Small intestine represents the most common site for the metastatic melanoma; however, it could be found anywhere in the gastrointestinal tract. Intussusception is a rare cause of intestinal obstruction in adults compared to children. In 90% of the cases, the underlying cause can be found, and in 65% of the cases, intussusception is caused by the neoplastic process. The majority of the neoplasms are benign, and about 15% are malignant. Metastatic melanoma is one of the most common metastatic malignancies to the gastrointestinal tract; however, the premortem diagnosis is rarely made. Here, we report an uncommon clinical presentation of metastatic melanoma causing intussusception in an 80-year-old man. This diagnosis should be considered in a differential diagnosis in any patient who presents with gastrointestinal symptoms and a history of melanoma.

## 1. Introduction 

The gastrointestinal (GI) tract is a common site of metastasis from malignant melanoma, but reports of related small bowel intussusceptions are rare [1]. Herein, we report a case of intussusception caused by metastatic malignant melanoma.

## 2. History and Operative Findings 

The patient is an 80-year-old man with a history of coronary artery disease, status post multiple stents requiring the use of Plavix and aspirin, cardiomyopathy with automatic cardioverter defibrillator implantation, gastroesophageal reflux disease, gastritis, hyperlipidemia, type 2 diabetes mellitus, and multiple resections for malignant melanoma from the left neck and right thigh. The patient had sentinel lymph node biopsy, which was found to be positive. He then had adjuvant radiation therapy. In 2011, the patient presented with gastrointestinal bleeding. He had previous GI bleeds thought to be related to GERD and gastritis. The initial endoscopy ruled out the upper and lower GI as a source of bleeding. However, a capsule endoscopy evaluation showed bleeding beyond the duodenum. The patient underwent another endoscopy where a jejunal tumor was found and biopsies were taken. The pathology report confirmed the diagnosis of malignant melanoma. The patient underwent a surgical resection where an area of small bowel intussusception was visualized.

## 3. Pathology

**Figure 1:**
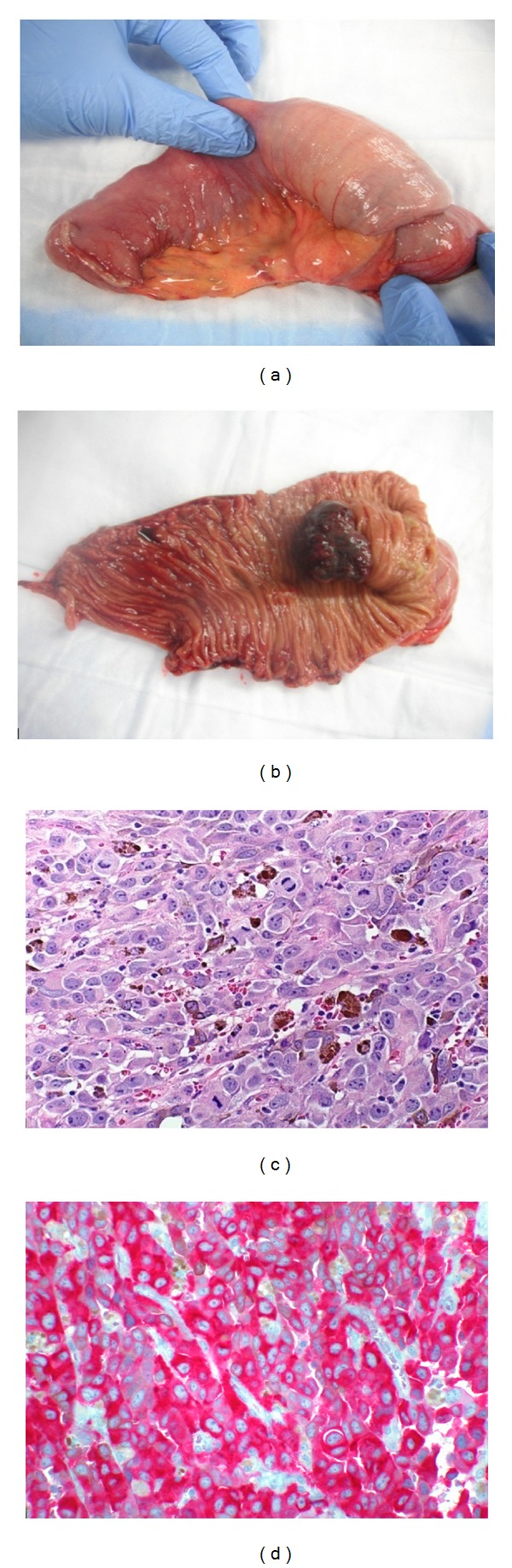
(a) Intussusception of the small bowel. (b) Exophytic pigmented mass. (c) Microscopic picture of the mass showing epithelioid cells with prominent nucleoli, brown pigment, and multiple mitotic figures (20 hpf). (d) Tumor cells are positive for Melan A (20 hpf).

A 19 cm segment of small intestine was resected which showed an area of intussusception (Figure 1(a)). The specimen was opened to reveal an exophytic polypoid gray-purple mass measuring 4 × 3 × 1.5 cm causing the telescoping of the bowel segment (Figure 1(b)). Microscopically, the polypoid mass was composed of epithelioid cells with eosinophilic cytoplasm, large nuclei, and prominent nucleoli. Melanin pigment was abundant (Figure 1(c)). The tumor involved the mucosa and submucosa and extended deeply into, but not through, the muscularis propria. Immunohistochemically, the tumor cells were positive for S100, HMB 45 and Melan A (Figure 1(d)) supporting the diagnosis of metastatic malignant melanoma.

## 4. Discussion 

Intussusception is a rare condition in the adult, in comparison to the pediatric patient group. In contrast to childhood intussusception, where 90% of the cases are idiopathic, adult intussusception has an underlying cause in 90% of the cases, 65% of which are due to neoplastic process [2]. Most of these tumors are benign; however, approximately 15% are malignant, mostly metastatic, with malignant melanoma being by far the most common [3]. Despite this, few cases of small bowel intussusceptions due to metastatic melanoma have been reported in the medical literature [1].

Malignant melanoma has a high metastatic capacity. Metastatic melanoma to the GI tract accounts for one third of all metastases to that region with small intestine being the most common site [4, 5]. The recorded incidence of GI metastases of melanoma in autopsy series reaches up to 60% [9]. However, only 2% to 4% of patients with malignant melanoma are clinically diagnosed with GI metastases [5, 6]. The symptoms include gastrointestinal bleeding, obstruction, abdominal pain, nausea and vomiting, and weight loss [7, 8]. The risk factors for malignant melanoma spread to the GI tract include superficial spreading melanoma, axial primary tumor, a Clark level III or IV, high degree of histologic, regression, ulceration, and high mitotic rate [1, 9, 10]. The interval between the diagnosis of primary malignant melanoma and clinical manifestation of GI metastases ranges from 2 to 180 months [11, 12]. This should prompt a careful clinical followup of patients with a history of high-risk melanoma who present with GI symptoms.

Herein, we report an uncommon clinical presentation in adults, intussusception, with an underlying pathology, metastatic melanoma to GI tract, that is a rarely diagnosed premortem. This diagnosis should be considered in any patient who presents with gastrointestinal symptoms and a history of malignant melanoma.
